# Topologization of β-antimonene on Bi_2_Se_3_ via proximity effects

**DOI:** 10.1038/s41598-020-71624-4

**Published:** 2020-09-03

**Authors:** K. Holtgrewe, S. K. Mahatha, P. M. Sheverdyaeva, P. Moras, R. Flammini, S. Colonna, F. Ronci, M. Papagno, A. Barla, L. Petaccia, Z. S. Aliev, M. B. Babanly, E. V. Chulkov, S. Sanna, C. Hogan, C. Carbone

**Affiliations:** 1 Institut für Theoretische Physik and Center for Materials Research (LaMa), Justus-Liebig-Universität Gießen, Heinrich-Buff-Ring 16, 35392 Gießen, Germany; 2 Istituto di Struttura Della Materia, Consiglio Nazionale Delle Ricerche, 34149 Trieste, Italy; 3 Ruprecht Haensel Laboratory, Deutsches Elektronen-Synchrotron DESY, 22607 Hamburg, Germany; 4 Istituto di Struttura Della Materia, Consiglio Nazionale Delle Ricerche, Via del Fosso del Cavaliere 100, 00133 Roma, Italy; 5 Dipartimento di Fisica, CS, Università Della Calabria, Via P. Bucci, 87036 Arcavacata di Rende, Italy; 6 Elettra Sincrotrone Trieste, Strada Statale 14 km 163.5, 34149 Trieste, Italy; 7 Azerbaijan State Oil and Industry University, AZ1010 Baku, Azerbaijan; 8 Institute Catalysis and Inorganic Chemistry, Azerbaijan National Academy of Science, AZ1143 Baku, Azerbaijan; 9 Departamento de Fisica de Materiales, UPV/EHU, 20080 Donostia-San Sebastian, Basque Country, Spain; 10 Donostia International Physics Center (DIPC), P. de Manuel Lardizabal 4, 20018 San Sebastián, Basque Country, Spain; 11 Saint Petersburg State University, 198504 Saint Petersburg, Russia; 12 Institute of Strength Physics and Materials Science, Russian Academy of Sciences, 634021 Tomsk, Russia

**Keywords:** Electronic properties and materials, Surfaces, interfaces and thin films, Topological insulators

## Abstract

Topological surface states usually emerge at the boundary between a topological and a conventional insulator. Their precise physical character and spatial localization depend on the complex interplay between the chemical, structural and electronic properties of the two insulators in contact. Using a lattice-matched heterointerface of single and double bilayers of β-antimonene and bismuth selenide, we perform a comprehensive experimental and theoretical study of the chiral surface states by means of microscopy and spectroscopic measurements complemented by first-principles calculations. We demonstrate that, although β-antimonene is a trivial insulator in its free-standing form, it inherits the unique symmetry-protected spin texture from the substrate via a proximity effect that induces outward migration of the topological state. This “topologization” of β-antimonene is found to be driven by the hybridization of the bands from either side of the interface.

## Introduction

Ever since the discovery of the importance of topology in condensed matter physics^1^, it has been demonstrated that the assembly of materials having different band structure topologies^2^ gives rise to a plethora of novel physical phenomena. The transformation of a topological insulator (TI) into a superconductor^3–5^, ferromagnetic TI^6^, or the appearance of Majorana fermions have been predicted^3^ and reported^7^. Topology has been invoked to explain the adsorbate/substrate resulting ferromagnetism^8–10^. Even graphene, in contact with a TI, shows effects related to spin–orbit coupling (SOC)^11,12^. These phenomena have been recognized as manifestations of a general ”topological proximity effect”.

A conventional insulator (CI) and a TI, although sharing similar electronic structure in the bulk, at their interface feature a gap closing due to the different topological invariants^2^. This process typically occurs via the emergence of metallic topological surface states (TSS), when spatially moving from the CI to the TI. In this paper we have investigated the behavior of the TSS that generally arise at the interface between the two insulators. The localization of the TSS can undergo a shift from the TI to the first layers of the CI, it may stay put at the interface, or it can move back inside the TI^13^. In other words, if a TSS migration arises at the interface, it can be the result of a “topologization” of the CI or a “trivialization” of the TI^9,14–19^.

Gap size, SOC strength, work function difference, and relative thickness^20^ of the CI and TI layers contribute to determine the properties of the resulting interface, as they drive the hybridization among atomic orbitals at the two sides of the interface. Structural aspects are also important: huge changes in the electronic structure have been predicted^21,22^ and experimentally reported as being due to lattice strain and to the relative orientation of the two lattices^23^. A step forward has been made in the case of β-antimonene (i.e. the buckled honeycomb 2D allotrope of Sb) grown on bismuth selenide, where a perfect match at the interface is obtained with single orientation and negligible strain^24–26^. A single, buckled β-antimonene sheet will be hereafter referred to as a bilayer (BL).

The β-antimonene/Bi_2_Se_3_ hetero-structure constitutes an archetypal system for studying these phenomena. Experimental evidence of a TSS migration has only been explicitly reported at the Bi/TI interface^27,28^ where the authors agree on the “topologization” of the bismuthene (i.e. honeycomb-like Bi) overlayer. However, one bilayer (BL) bismuthene hosts one-dimensional (1D) edge states^29,30^ typical of the quantum spin Hall phase (QSH), so that when deposited on top of a topological insulator, both 1D and 2D edge/surface states spatially coexist^27^. In contrast, 1 BL and 2 BL of β-antimonene are trivial insulators^31^ while showing an important SOC as in the case of bismuth. This results in a simpler electronic structure, whose spin texture can be understood by atomistic calculations. This is of paramount importance in applications exploiting the electron spin, as tailoring the CI/TI spin textures can boost the realization of spintronics devices.

In the following we report on the atomic and electronic structures of atomically ordered β-antimonene layers on Bi_2_Se_3_(0001). These systems have been examined by scanning tunneling microscopy and spectroscopy (STM/STS), angle-resolved photoemission spectroscopy (ARPES) and ab initio calculations within the density functional theory (DFT). STM images reveal the formation of ordered single and double BL of β-antimonene on Bi_2_Se_3_, while STS of the unoccupied states reveal fingerprints of emergent topological bands. In both systems, ARPES measurements display well-defined dispersing bands, in good agreement with the calculated band structures. Hybridization between β-antimonene and Bi_2_Se_3_ determines the spin pattern of surface and interface electronic states. The formation of Dirac-cone surface-features demonstrates the topological character that β-antimonene inherits from the substrate via the proximity effect.

## Results and discussion

The main issue with van der Waals (vdW) hetero-structures, is the difficulty in isolating a *single* overlayer phase in order to unambiguously characterize its electronic structure. Antimonene grown on Bi_2_Se_3_ forms at least two different allotropes: a puckered rectangular (α) and a buckled hexagonal (β) phase. We demonstrated a novel procedure for synthesizing large areas of lattice-matched β-antimonene on Bi_2_Se_3_ by means of a controlled structural transition from the α to the β phase^25,26^. This results in a perfectly matched and oriented β-antimonene single phase on Bi_2_Se_3_.

**Figure 1:**
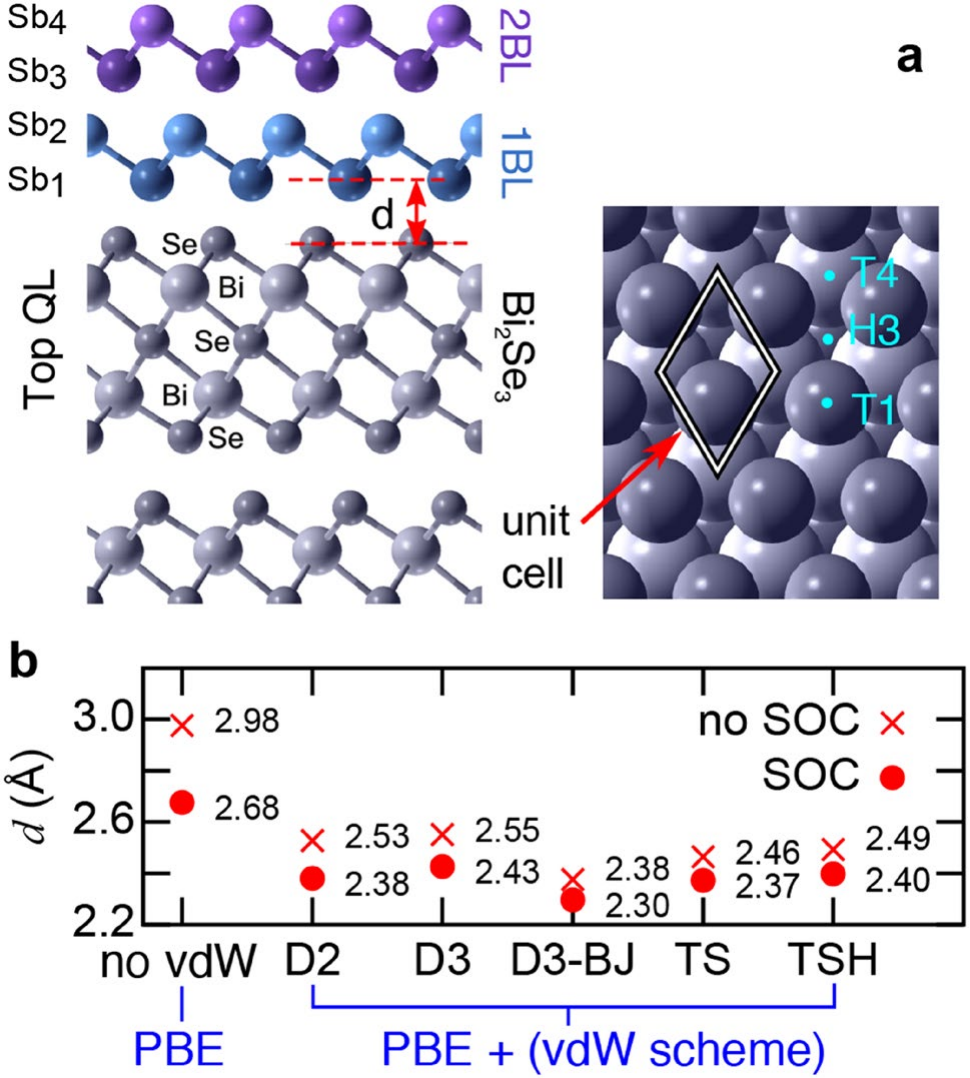
Structural properties of the β-antimonene/Bi_2_Se_3_ hetero-junction. (**a**) Schematic structure of the β-antimonene/Bi_2_Se_3_ interface for different coverages. The surface unit cell and interlayer spacing *d* are indicated, as well as positions of the T1 (atop Se), H3 (hollow), and T4 (atop Bi) sites. In the 1 BL case, the Sb_2_ (Sb_1_) atom is adsorbed over the T4 (H3) site. The 2 BL layer follows the bulk stacking order, so that Sb_3_ lies in the 1 BL hollow site. (**b**) Dependence of *d* for the 1 BL case on the vdW implementation (see text for details) and SOC.

The resulting hetero-junction is shown in Fig. 1a. Each β-antimonene BL is composed of two Sb planes (Sb_1_ and Sb_2_ for the BL in contact with the substrate, Sb_3_ and Sb_4_ for the outer BL) arranged to form a buckled honeycomb structure. For the 1 BL case, we tested the influence of various vdW treatments on the DFT-calculated inter-layer spacing *d,* between β-Sb and Bi_2_Se_3_, and report the results in Fig. 1b. The inclusion of any vdW treatment leads to consistently smaller values of *d* with respect to a pure PBE calculation. As a result, calculations neglecting vdW in determining the hetero-structure geometry may underestimate wave-function hybridization and lead to strong misalignments of β-antimonene and Bi_2_Se_3_ bands. Figure 1b also demonstrates the importance of accounting for SOC when computing the geometry. SOC reduces the inter-layer spacing *d* and has thus a similar effect as the vdW term. Since the semi-empirical D2 method^32^ yields results in good agreement with heavier *ab-initio* vdW flavours, and correctly describes the bulk Bi_2_Se_3_ geometry, we adopt henceforth the PBE + SOC + D2 geometries.

**Figure 2:**
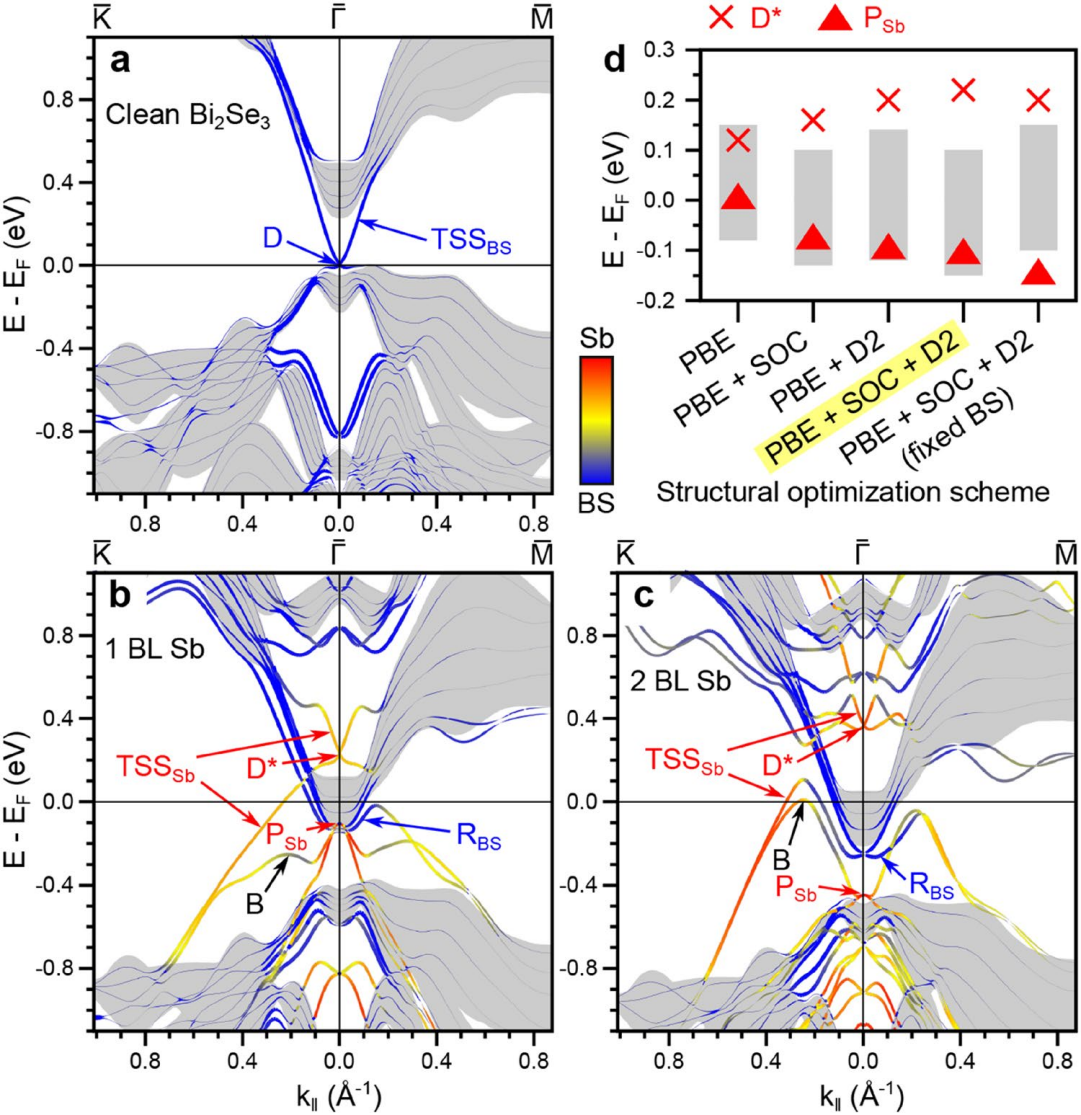
Computed band structures. (**a**) Bi_2_Se_3_, (**b**) 1 BL and (**c**) 2 BL of β-antimonene on Bi_2_Se_3_ along ${\bar{\text{K}}}-{\bar{\Gamma}}-{\bar{\text{M}}}$ at the PBE-SOC-D2 geometry. The blue-to-red color gradient indicates the relative projection onto the BS front surface and Sb layers, respectively. Solid grey areas are the projected bulk bands. **d** Energy position at ${\bar{\Gamma}}$ of TSS_Sb_ (crosses) and P_Sb_ (triangles) for the 1 BL case using slab geometries computed with different schemes. The grey columns indicate the projected bulk conduction band at ${\bar{\Gamma}}$.

Band structures of clean Bi_2_Se_3_, and with 1BL and 2BL of β-antimonene are reported in Fig. 2a–c along the ${\bar{\text{K}}}-{\bar{\Gamma}}-{\bar{\text{M}}}$ symmetry lines. The color scale of the bands indicates their spatial origin by means of projection onto atomic orbitals of (red) β-antimonene or (blue) Bi_2_Se_3_ surface (defined here as half the 6 QL slab). This allows to distinguish truly surface-localized states of Bi_2_Se_3_ from bulk states (projected bulk in gray). Orange/yellow lines indicate hybridized states of mixed β-antimonene/Bi_2_Se_3_ (Sb/BS) character. The Fermi level (E_F_) for both β-antimonene systems cuts the projected conduction band of bulk Bi_2_Se_3_, in contrast to the case of clean Bi_2_Se_3_, whose E_F_ is pinned at the Dirac point D of the topological surface state (TSS_BS_). This reflects a charge transfer from Sb to the substrate upon deposition. Similar calculations have been reported previously for 1 BL^14^ and 2 BL of β-antimonene^24^ on Bi_2_Se_3_. We refer the reader to these works for a more detailed discussion of the origin of the band dispersion changes. Some important quantitative differences with respect to our calculations are discussed below.

Several bands are visible within the projected bulk gap. For the clean surface (Fig. 2a), the dominant feature is TSS_BS_. The hetero-structures have more complicated band structures showing several hybridized bands for the single and double BLs. A topological surface state TSS_Sb_ with corresponding Dirac point D* is evident in both systems. This state has a mixed orbital character for 1 BL and is of almost pure Sb-character near ${\bar{\Gamma}}$ in the 2 BL system (in fact it is localized more strongly on the outermost Sb bilayer). The hybridization with Sb leads to modifications of the band dispersion around D*. For the 2 BL case, the effect of hybridization is so strong that the lower branch of the Dirac cone is unrecognizable. In both cases D* appears in the projected bulk local gap above E_F_, while the TSS_Sb_ are clearly distinguished within the absolute gap, below E_F_. A dispersing band B of mixed orbital character is found below TSS_Sb_ and merges with it at larger wave-vector (k_||_) values for both Sb coverages. A state of almost pure Sb character (P_Sb_) is found near the Bi_2_Se_3_ conduction band minimum for the 1 BL case, where also Rashba-split bands (R_BS_), mainly deriving from the substrate, appear beside and below the bulk conduction bands. Similar Rashba bands are obtained for the 2 BL case, while P_Sb_ is shifted near the valence band maximum.

Previous calculations of the 1 BL structure placed D* inside the Bi_2_Se_3_ conduction band and the apex of P_Sb_ inside the bulk gap^14^. We attribute this difference mostly to the omission of vdW interactions when optimizing the hetero-structure geometry. Figure 2d indicates the energetic position of P_Sb_ and D* at ${\bar{\Gamma}}$ for the 1 BL case, as a function of the computational scheme adopted during the geometry optimization. The grey shaded area indicates the projected bulk conduction band. In the case of pure PBE (no vdW treatment), both D* and P_Sb_ lie inside this band. Both points shift in energy, however, if vdW and/or SOC are included during the relaxation. Fixing the substrate geometry to that of bulk Bi_2_Se_3_ causes a strong misalignment of these bands with respect to the bulk bands (rightmost column). This stresses the importance of structural relaxation, dispersion and SOC in studies of such topologically-hybridized hetero-structures. Further modifications of the band structures are expected when quasi-particle corrections^33–35^ are considered.

**Figure 3:**
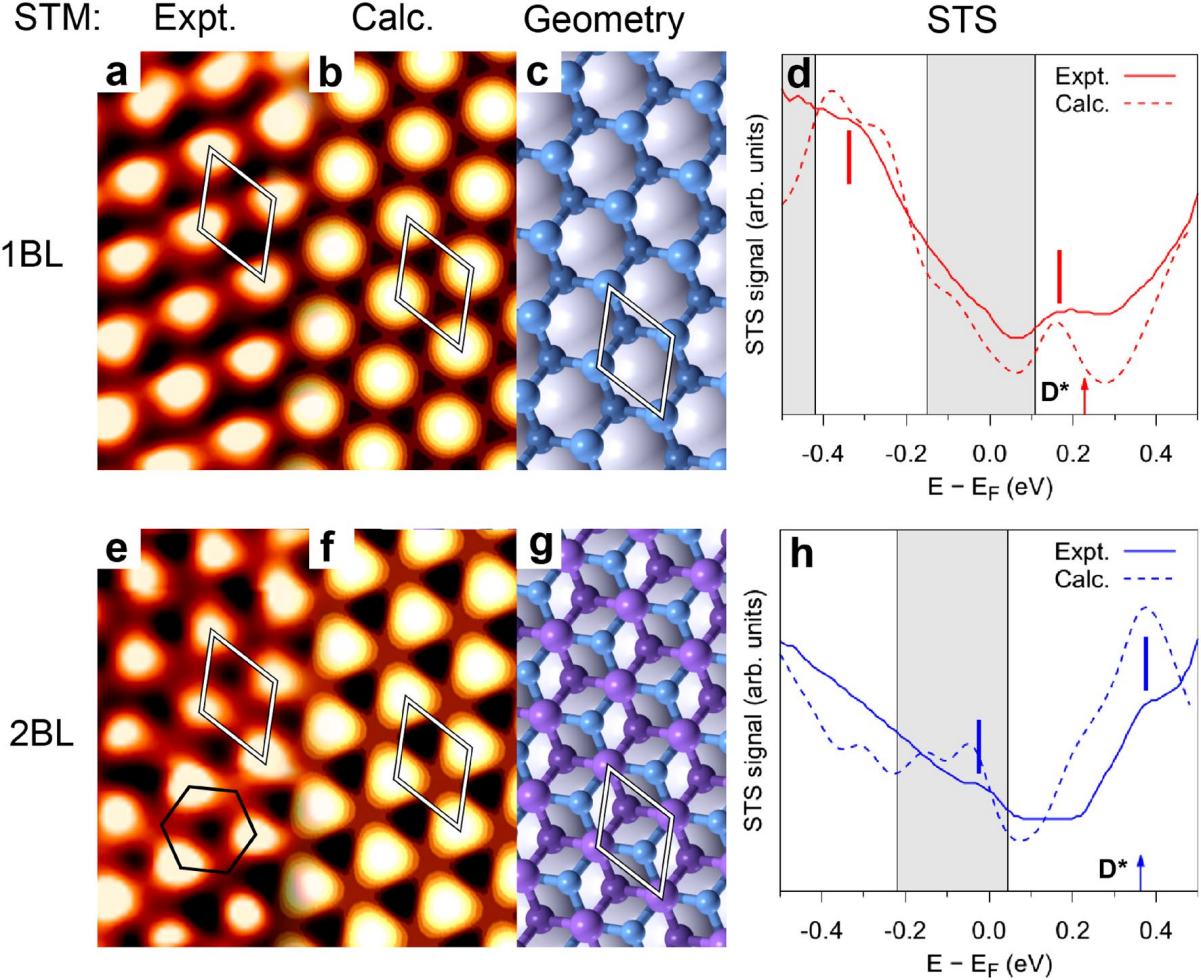
STM and STS measurements compared with theoretical calculations. The top and bottom rows refer to 1 BL and 2 BL β-antimonene/Bi_2_Se_3_, respectively. (**a,e**) 1.1 × 2.2 nm^2^ experimental STM images (V_bias_ =  + 0.2 V, I = 20 nA) and (**b,f**) simulated STM images (V_bias_ =  + 0.2 V) based on the optimized structure shown in (**c,g**). (**d,h**) Experimental normalized (dI/dV)/(I/V) STS spectra and computed LDOS. Grey regions indicate extent of projected bulk bands at ${\bar{\Gamma}}$ point; D* indicates the calculated position of the emergent Dirac point in each case. Vertical bars represent the main experimental features (see text for discussion).

Figure 3 shows (a) the STM image, (b) its DFT simulation, (c) the ball and stick model (top view, unit cell is highlighted) and (d) the experimental and simulated STS spectra for 1 BL β-antimonene on Bi_2_Se_3_. The experimental and simulated STM images are in very good agreement and clearly show a hexagonal pattern determined by the upper Sb atoms (Sb_2_ in Fig. 1a). An equivalent comparison for the 2 BL case is reported in Fig. 3e–h. Although the buckled atomic structure of the topmost BL is almost identical to that of the 1 BL case^26^, the STM image is quite different, suggesting an important LDOS contribution to the STM images. The image is dominated by bright spots associated with the topmost Sb atoms (Sb_4_), but in this case a clear apparent buckled honeycomb pattern is observed in both measured and computed STM images. Similar simulated images are obtained for different positions of E_F_ from − 0.15 eV to + 0.15 eV.

The simulated STM image in panel (f) reveals that the apparent buckling does not derive from the geometrical buckling of the topmost Sb BL. Indeed, the Sb atom (Sb_3_) in the unit cell in Fig. 3g is imaged as a hollow in both the experimental and simulated STM images. From analysis of the integrated local density of states (LDOS), we find that TSS_Sb_ strongly contributes to the 1 BL and 2 BL STM images reported in Fig. 3a,e (this is particularly true for the 1 BL case). The apparent buckling observed in the 2 BL STM images is actually determined by the shape and decay profile of the TSS_Sb_ wave-function.

Figure 3d,h shows a comparison between the measured normalized differential conductance dI/dV/(I/V) spectra for the β-antimonene layers and the computed LDOS. The grey shaded areas indicate the projected bulk bands at the ${\bar{\Gamma}}$ point. The main experimental features are reproduced in the computed curves, such as the peaks at about − 0.32 eV and + 0.18 eV in the 1 BL case and the shoulders at − 0.02 eV and + 0.38 eV in the 2 BL spectra. Also evident is a “gap” in the 2 BL spectrum from about 0.05 to 0.23 eV that reflects a well-resolved minimum in the calculated data. The computed 1 BL curve in Fig. 3d shows a minimum very close to the position of the calculated D* (0.23 eV): the alignment is not perfect due to the imposed broadening and nearby presence of the peak at 0.15 eV. Accordingly, the relative experimental curve shows a clear dip at the same energy, similarly to Sb on copper^36^. The calculated 2 BL STS curve does not show a minimum in correspondence of the D* energy, since the LDOS is dominated by peaks of similar energy. A comparison with the computed band dispersions reveals that the four peaks and shoulders in the STS derive from key surface states of the β-antimonene/Bi_2_Se_3_ hetero-structures. The peaks at + 0.18 eV (1 BL) and + 0.38 eV (2 BL) are fingerprints of the emergent topological surface states TSS_Sb_, which exhibit strong curvature or even flattening in the vicinity of D*. These features lie inside a gap in the projected bulk band structure as shown in Fig. 2b,c. In Ref.^24^ a sharp peak in the STS spectrum of 2BL β-antimonene/Bi_2_Se_3_ at about 0.45 eV is attributed to a stationary point in the substrate conduction bands (their calculations place D* at 0.28 eV). However, their peak is well consistent with the sharp feature found at + 0.38 eV in our computed LDOS arising from TSS_Sb_ in the vicinity of D*. This discrepancy may be due to the neglect of vdW interactions in Ref.^24^. The shoulders at − 0.32 eV (1 BL) and − 0.02 eV (2 BL) derive from the B bands which reverse slope at around 0.25 Å^−1^ on either side of the ${\bar{\Gamma}}$ point.

**Figure 4:**
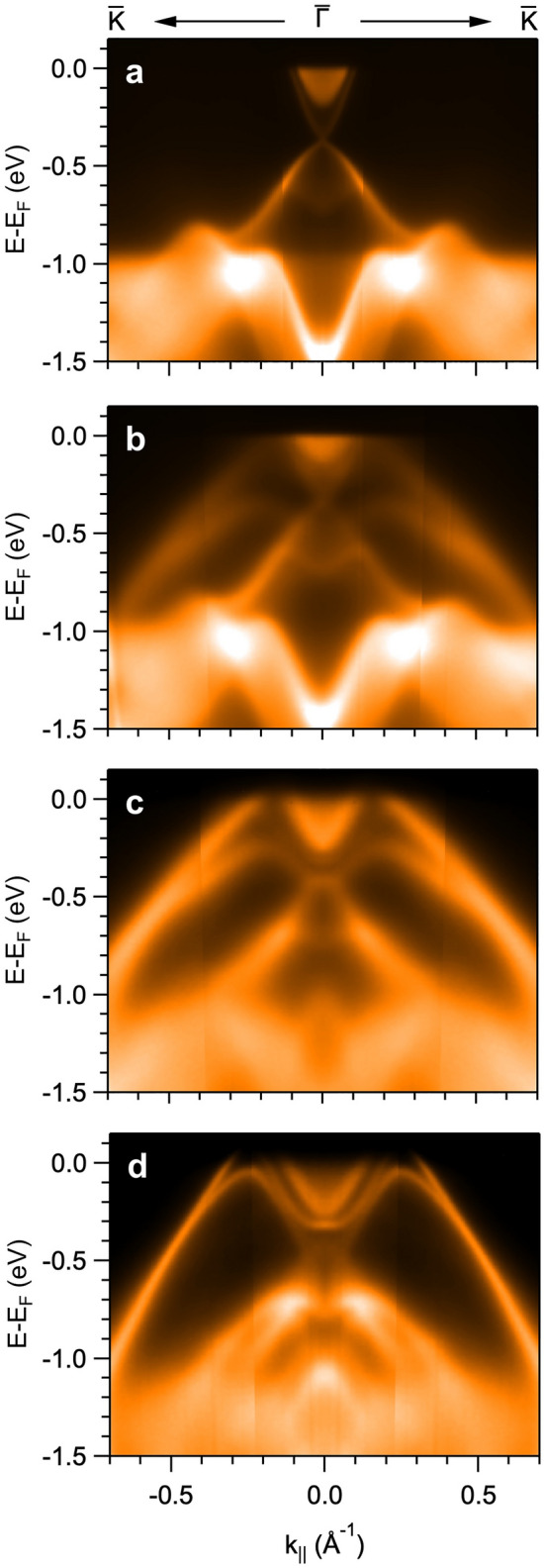
Experimental band structure. ARPES spectra measured with 20 eV photon energy along ${\bar{\text{K}}}-{\bar{\Gamma}}-{\bar{\text{K}}}$ of (**a**) Bi_2_Se_3_, (**b**) 0.6 BL, (**c**) 1.1 BL and (**d**) 2 BL of β-antimonene/Bi_2_Se_3_.

Figure 4 displays ARPES spectra of Bi_2_Se_3_ and β-antimonene/Bi_2_Se_3_ for different Sb coverages along the ${\bar{\Gamma}} -{\bar{\text{K}}}$ direction. Clean Bi_2_Se_3_ (Fig. 4a) shows a pair of linearly dispersing surface states near E_F_ that are identified as TSS_BS_. D occurs at − 0.34 eV, in line with the results summarized in Ref.^37^. Other intense bulk and surface features of Bi_2_Se_3_ are observed at larger binding energies. With the formation of 1 BL and 2 BL β-antimonene, new features appear in the Bi_2_Se_3_ band gap, while the substrate bands are attenuated and shifted to lower energies by the above discussed charge transfer process. Figure 4b,c shows data corresponding to Sb coverages below (0.6 BL) and slightly above (1.1 BL) the completion of 1 BL, respectively. In both cases two dispersing bands are observed between E_F_ and − 1 eV. They split upon approaching the zone center: one branch crosses E_F_; the other reverses its dispersion after reaching about − 0.3 eV. Figure 4d shows ARPES spectra for a full 2 BL β-antimonene. The dispersion of the Sb-induced features becomes sharper with respect to the 1 BL. Similarly to the 1 BL case, two bands are degenerate far from $\overline{\Gamma}$. Close to the zone center, one band crosses E_F_ whereas the other one bends to deeper energy. Another feature characteristic of the 2 BL system, and weakly visible in Fig. 4c, is observed at $\overline{\Gamma}$ with a maximum at − 1 eV.

ARPES measurements on systems comprising few BL antimonene are available in the literature. Lei et al*.*^38^ investigated sub-BL coverage. In Ref.^24^, the ARPES spectra reported for 2BL actually correspond to a mixture of islands of different heights. In all cases, the samples are grown at RT and exhibit the presence of several allotropic phases^25,26^. The features described here can be considered as true ARPES reference spectra for a *single* β-antimonene phase for 1 BL and 2 BL at *full* coverage.

**Figure 5:**
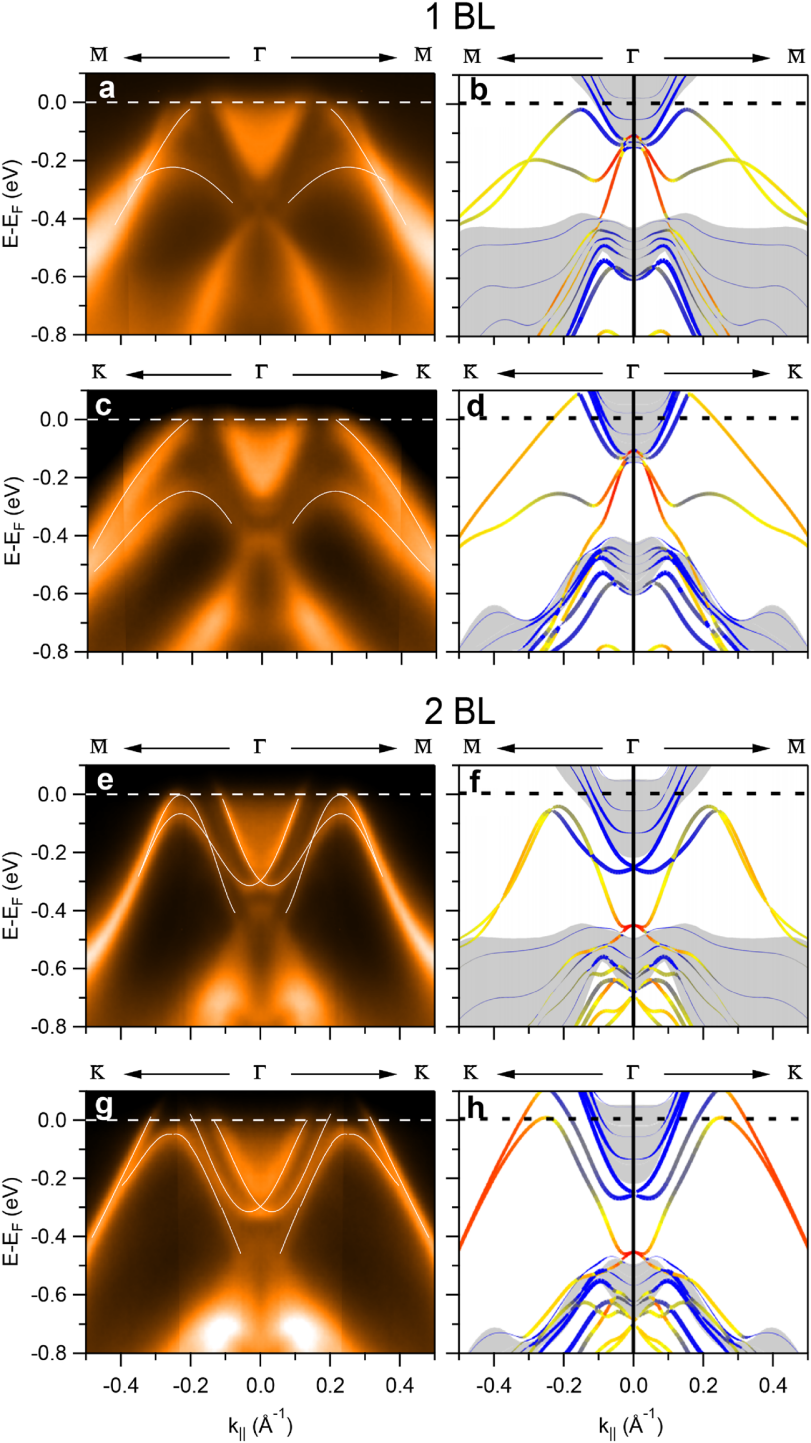
ARPES spectra and orbital-projected band structure. (**a**–**d**) 1 BL β-antimonene along the (first row) ${\bar{\text{M}}}-{\bar{\Gamma}}-{\bar{\text{M}}}$ and (second row) ${\bar{\text{K}}}-{\bar{\Gamma}}-{\bar{\text{K}}}$ directions. (**e–h**) Same as (**a–d**) for 2 BL β-antimonene.

Figure 5 compares the experimental and calculated band structures of 1 BL and 2 BL on Bi_2_Se_3_ along ${\bar{\text{M}}}{-}{\bar{\Gamma}}{-}{\bar{\text{M}}}$ and ${\bar{\text{K}}}{-}{\bar{\Gamma}}{-}{\bar{\text{K}}}$. In spite of the lack of quasi-particle corrections, and allowing for some misalignment of E_F_, the overall agreement is rather good for 1 BL (Fig. 5a–d). The upper branch derives from the lower part of the TSS_Sb_ band, while the lower branch can be identified with the band B. Both bands have a hybrid Sb/Bi_2_Se_3_ character. The Sb-related state P_Sb_ with maximum at − 0.10 eV at ${\bar{\Gamma}}$ cannot be clearly distinguished in the experiment, however. This can be attributed to the overlap with the substrate conduction band and to matrix element effects: according to DFT, the state has purely p_x_, p_y_ character and thus a low cross section in the photoemission experiments with p-polarized light in off-normal incidence.

Bands with an apparently similar dispersion in the proximity of E_F_ were observed also for Sb layers grown on other TIs^38,39^ and attributed to 1 Sb BL of unknown structure. However, the slopes of the bands for Sb layers on Sb_2_Te_3_ and Bi_2_Te_3_ are very different from the ones reported here and hardly compatible with the theoretical prediction. Theory predicts similar group velocities near E_F_ for the TSS_Sb_ bands on Sb_2_Te_3_ (1.4 eV × Å), Bi_2_Te_3_ (1.7 eV × Å) and Bi_2_Se_3_ (1.3 eV × Å) substrates^14^. The group velocities measured for the TSS_Sb_ bands in Ref.^39^ were 3.1 eV × Å on Sb_2_Te_3_ and 2.5 eV × Å on Bi_2_Te_3_, i.e. almost twice larger than expected. In our DFT calculations we find a group velocity of approximately 1.5 eV × Å, which is in perfect agreement with the one determined by the ARPES data in Fig. 5 (1.6 eV × Å).

Figure 5e–h shows the comparison of experimental and calculated band structure of 2 BL β-antimonene on Bi_2_Se_3_. The overall agreement is even better than for the 1 BL case. The larger splitting of the upper and lower branches along ${\bar{\text{K}}}{-}{\bar{\Gamma}}{-}{\bar{\text{K}}}$ than along ${\bar{\text{M}}}{-}{\bar{\Gamma}}{-}{\bar{\text{M}}}$ is well reproduced. Moreover, the E_F_ crossing of these bands is also consistent. Only one band crosses E_F_ along ${\bar{\text{K}}}{-}{\bar{\Gamma}}{-}{\bar{\text{K}}}$, while no crossing is seen along ${\bar{\text{M}}}-{\bar{\Gamma}}-{\bar{\text{M}}}$. From the orbital-projected band structure one can again identify the two strongly dispersive bands with the emergent TSS_Sb_ band and the mixed origin of the B band. Furthermore, the band crossing near − 0.3 eV at ${\bar{\Gamma}}$ (also present albeit less well resolved in the 1BL spectra) can be clearly interpreted as due to Rashba-split bands. According to the calculations, these bands are generated by the interface with β-antimonene but localized in the topmost Bi_2_Se_3_ QLs. Their orbital character is very different from that of the original TSS_BS_, being derived from the bulk conduction band. The Rashba splitting is caused by local dipole moments between QLs associated with the charge transfer from the β-antimonene to the substrate^40,41^.

We complete our analyses by considering the topological character of both systems as revealed by DFT. We verified that the free-standing 1 BL and 2 BL β-antimonene films are topologically trivial insulators by computing the *Z*_*2*_ topological invariants (see Supplementary Information for details) following the parity method of Fu and Kane^42^. This finding is consistent with previous calculations that predict a trivial-topological crossover to a quantum spin Hall phase for layers of at least 4 BLs^31^. We also note that an odd number of bands cross the fundamental gap along ${\bar{\Gamma}}{-}{\bar{\text{M}}}$, in both cases due to the single TSS_Sb_ branch that connects the bulk valence band to the bulk conduction band^2,43^.

**Figure 6:**
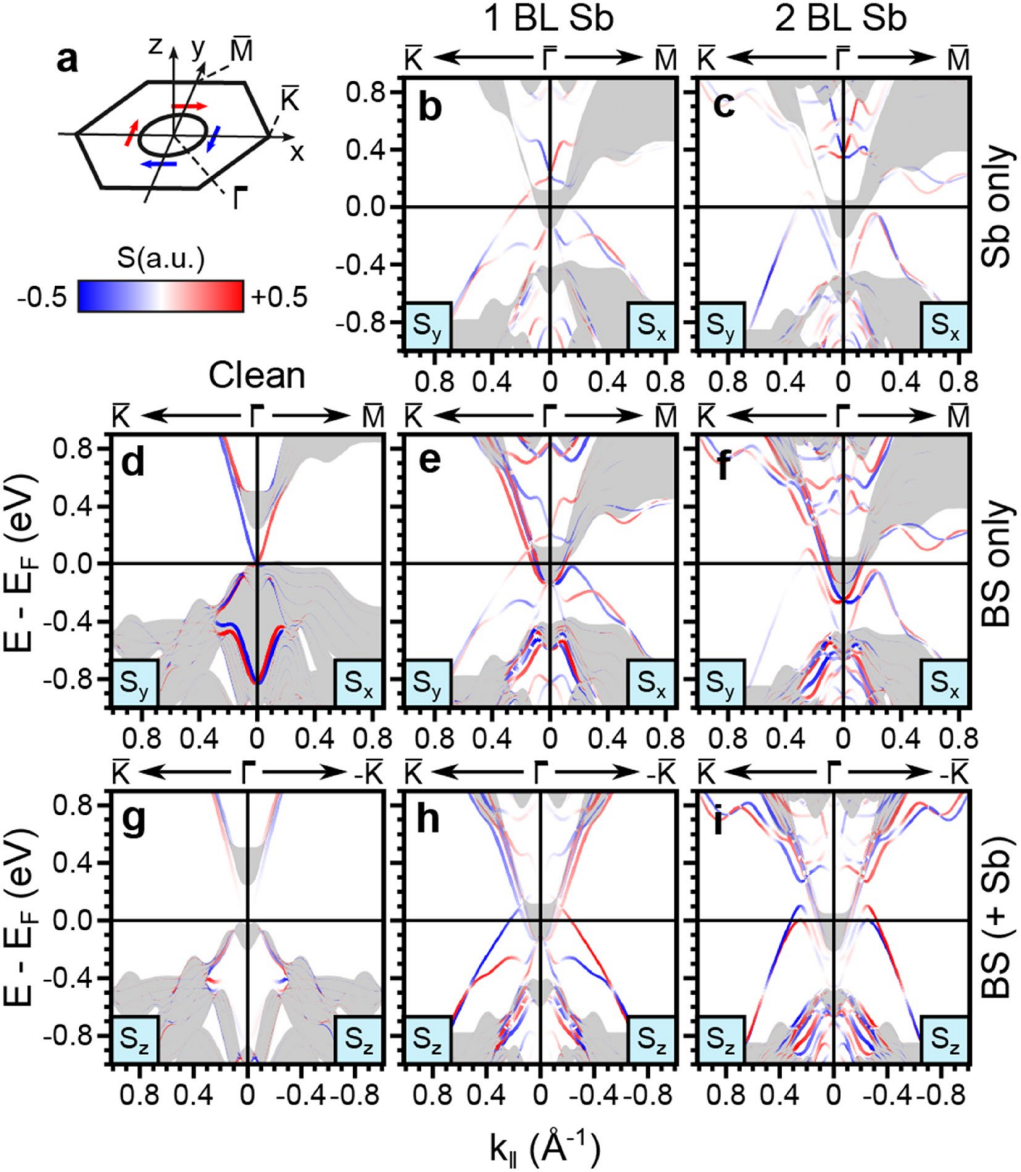
Computed spin texture. (**a**) Brillouin zone geometry. (**b–i**) Spin textures of Bi_2_Se_3_ (left column) and additional 1 BL β-antimonene (centre column) and 2 BL β-antimonene (right column). (**b–f**) In-plane S_x_ and S_y_ components projected on the β-antimonene layer (top row) and the top QL (middle row). (**g–i**) S_z_ component, for selected spatial projections. Spin up and spin down channels are represented in blue and red, respectively.

The calculated spin texture of the clean Bi_2_Se_3_ and β-antimonene/Bi_2_Se_3_ hetero-structures is reported in Fig. 6. Figure 6b-f displays the S_x_ and S_y_ components of the spin expectation value along ${\bar{\text{K}}}{-}{\bar{\Gamma}}{-}{\bar{\text{M}}}$ (i.e. perpendicular to *k*_*x*_ and *k*_*y*_, as defined in Fig. 6a), projected on the β-antimonene layers (top row) and topmost Bi_2_Se_3_ QL (middle row), respectively. Zero polarization is found along the direction parallel to *k* [See Fig. S3 and S4 of Supplementary Information]. Figure 6g–i shows the out-of-plane component of the spin expectation value S_z_ along ${\bar{\text{K}}}-{\bar{\Gamma}}-{\bar{\text{K}}}$. While S_z_ is practically zero in pure Bi_2_Se_3_, it becomes strong for the hetero-structures, even close to the ${\bar{\Gamma}}$ point for some bands. This may derive from a hexagonal warping effect induced by the β-antimonene layer^44^ or from considerable orbital momentum contributions^45^. Several bands such as the band of mixed Sb/BS character labeled B, as well as TSS_Sb_ in the 1 BL case, show *k* dependent rotations in S_y_. The B band in particular has a dominant S_z_ component. In contrast, the Rashba-like character of R_BS_ is confirmed by the spin texture which is completely confined to the substrate and lies fully within the surface plane (zero S_z_ component). The second band crossing lying in the projected local bulk gap was clearly detected in the experiment (see around − 1.1 eV in Fig. 4d) which likely corresponds to the Rashba-split bands seen here at − 0.8 eV in the calculation. These latter bands have strong Sb character and thus have S_z_ = 0 only around the ${\bar{\Gamma}}$ point.

Spin analysis along the ${\bar{\text{K}}}{-}{\bar{\Gamma}}{-}{\bar{\text{K}}}$ direction (see Figs. S3 and S4 of Supplementary Information) confirms the helical nature of TSS_Sb_. The 1 BL bands show a similar spin pattern to that of the substrate, wherein the spin magnetization density axis is locked perpendicular to *k*. For the 2 BL case, the spin polarization of TSS_Sb_ within the surface plane shows the same spin-momentum locking, at least in a narrow range around ${\bar{\Gamma}}$ well above the E_F_. The spin polarization of the TSS decreases from the center to the boundary of the Brillouin zone for the S_x_ and S_y_, while the opposite occurs for the S_z_. This is generally ascribed to the superposition with bulk projected states^46^. TSS_Sb_ thus displays the character of an emergent topologically protected state. By the comparison of the spin texture around the D (clean Bi_2_Se_3_) and D^*^ points (either 1 BL or 2 BL), we note that the spin texture associated with the original TSS persists following hybridization with Sb states. In contrast, TSS_BS_ is barely present in the Sb-covered surfaces (being hidden by the bulk valence band), implying that the state has lost its character (and topological protection) upon interfacing of the Sb layer.

**Figure 7:**
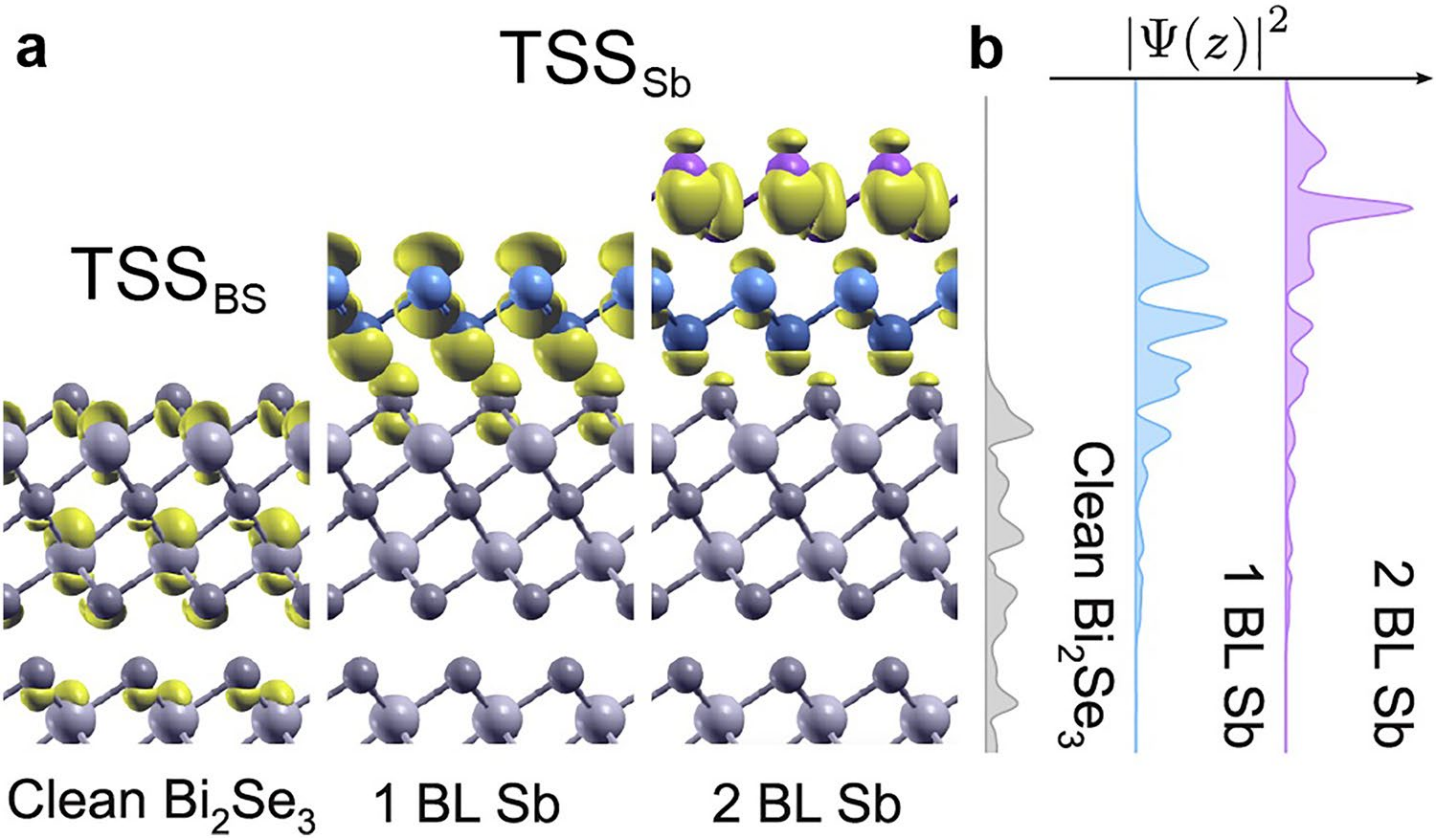
The real space character of TSS_BS_ and TSS_Sb_. (**a**) Isosurfaces of TSS_BS_ or TSS_Sb_ at the ${\bar{\Gamma}}$ point and (**b**) their planar-averaged profiles.

The spin texture of TSS_Sb_ derives mostly from the β-antimonene BL(s), although there is also a considerable contribution from the topmost QL in the 1 BL case, as expected from the orbital projection data (Fig. 2b,c). Figure 7a,b show TSS_BS_ and TSS_Sb_ in real space as isosurface plots of $|\overline{\uppsi }|$^2^ at the ${\bar{\Gamma}}$ point and as plane-averaged plots, respectively. These plots at the D and D* points demonstrate the change in the character and localization of the topological state as a function of the Sb coverage. All three states have a similar total extension perpendicular to the surface (10–15 Å). The TSS_BS_ state spans the whole topmost quintuple layer, as well as the first two atomic layers of the second QL, and derives from a mixture of Bi and Se orbitals. The TSS_Sb_ is distributed across and outside the β-antimonene in the 1 BL system, with some contribution from the substrate (especially Se p_z_ orbitals). Its depth profile is thus very similar to that of clean Bi_2_Se_3_ although it has clearly shifted towards the β-antimonene layer. The depth profile for 2 BL TSS_Sb_ is quite different, being more strongly localized at the outermost β-antimonene BL and with weaker contributions from lower Sb and Se atoms. The different orbital origin of the two TSS_Sb_ states conclusively explains the differences between their STM images in Fig. 3. The associated bands and spin texture around the D and two D* points (compare Fig. 6b,c,d) nonetheless remain strikingly similar.

These observations confirm that TSS_Sb_ and the Dirac point at D* are truly emergent features and constitute a manifestation of a proximity effect in which an adsorbed trivial insulator (β-antimonene) inherits the unique electronic properties of a TI substrate (Bi_2_Se_3_). In other words, the helical state floats to the top of the CI (β-antimonene) which acquires a topological character^47^. The hybridization with the substrate demonstrates that Bi_2_Se_3_ does not provide a platform for realizing free-standing β-antimonene, in spite of its vdW nature^26^. However, it opens up the possibility to harness the topological nature of the Bi_2_Se_3_ substrate along with its momentum-locked spin character.

## Conclusions

The preparation of large areas of single β-phase of antimonene on Bi_2_Se_3_ allowed to record unprecedently detailed ARPES and STM/STS reference spectra for 1BL and 2BL. Accurate quantum mechanical simulation including vdW and SOC have been performed to interpret the experimental data. The excellent agreement achieved with respect to the measured ARPES, STS, and STM data allows a consistent analysis of the experiments and provide a sound theoretical description of the underlying physical phenomena.

Our approach reveals a migration of TSS from the TI substrate to the outermost atomic layers of the CI. The migration occurs in a step-by-step manner as 1 and 2 BL of β-antimonene are adsorbed. In other words, the particular electronic character of the β-antimonene/Bi_2_Se_3_ hetero-structures makes it possible to successfully *extract* the TSS from the TI surface and transfer it to the topmost atomic layers of the topologically trivial insulator. This behavior confirms the process of CI “topologization” in the hetero-structure, as distinct from the predicted “trivialization” of the TI in MnSe/Bi_2_Se_3_^9^, or the absence of migration of the TSS as predicted theoretically in ZnS/Bi_2_Se_3_^13^. The spatially extended character of TSS in β-antimonene/Bi_2_Se_3_ demonstrates that it originates from the topology of bulk Bi_2_Se_3_, although orbital analysis reveals that it acquires considerable Sb character due to increasing hybridization. This origin appears to differ from previous suggestions^24^.

The TSS migration is facilitated by the perfect lattice match of β-antimonene/Bi_2_Se_3_. Other vdW hetero-structures with incommensurate interfaces, like α phase of antimonene on Bi_2_Se_3_^26^, could hinder such migration due to the different interface potential, orbital hybridization, and local geometry, leading to topological states with different dispersion and localization. Lattice matching is therefore crucial in the design of vdW hetero-structures with topological properties.

From a technological point of view, the β-antimonene/Bi_2_Se_3_ heterostructures considered here do not directly offer a route towards dissipationless currents, as bulk conduction bands open multiple scattering channels at E_F_. It might be feasible to tune the position of the D* points into the bulk fundamental gap by means of surface doping^48^ or external strain^49^. Yet, the strong perturbation of the lower Dirac cones towards a Rashba-like character may offer alternative possibilities for probing electronic excitations of topologically protected helical states via inverse photoemission^50^, two-photon photoemission^51,52^, or optical techniques^53,54^.

Finally, symmetry protection allows the migration of the TSS from a TI substrate to an adsorbate layer that is both elementally and structurally different. This includes the change in symmetry from the hexagonal Se layer to the honeycomb Sb, as well as the change from a passivated substrate to one with dangling bonds. This is relevant to all the cases where the surface of the TI is not suitable to an epitaxial growth or to a functionalization. Ultimately, this may open a novel route towards easier handling of materials made of TI interfaced with metal contacts for the next generation of devices.

## Methods

### Sample preparation, STM/STS and ARPES measurements

Antimony was sublimated on in-situ cleaved Bi_2_Se_3_ at RT with a deposition rate of 0.5 Å/min, followed by a prolonged annealing at 473 K^25^. The Sb coverage calibration was based on the known structural properties of the Ag_2_Sb surface alloy on Ag(111), which presents a $(\sqrt{3} \times \sqrt{3})R30^\circ$ reconstructed surface^55^. The structural ordering of the sample was confirmed by low-energy electron diffraction (LEED). STM images were measured by using an Omicron LT-STM instrument operating at 80 K. An electrochemically etched W wire was used as STM tip after electron bombardment cleaning in UHV. The STM scanner was calibrated measuring the clean Bi_2_Se_3_(0001) surface. The bias voltage is referred to the sample, hence positive (negative) bias corresponds to empty (filled) states. STS measurements have been acquired at 80 K using a lock-in amplifier with gap modulation at 5 kHz and 10 mV peak-to-peak amplitude. The STS spectra were collected on a 30 × 30 nm^2^ image on a grid of 80 × 80 points. The reported spectra were obtained by averaging the normalized differential conductance curves (dI/dV)/(I/V) for 1 and 2 BL of β-antimonene. Photoemission experiments were performed at the VUV-Photoemission and BaDElPh^56^ beamlines of Elettra (Trieste, Italy). ARPES measurements were conducted at 80 K, with an angular resolution better than 0.3° and energy resolution of 30 meV.

### Calculation method

DFT calculations were performed using the Vienna ab initio simulation package (VASP)^57,58^. Plane waves (kinetic energy cutoff of 400 eV) and projector augmented wave (PAW) pseudopotentials [(15 valence electrons for Bi (electronic configuration 5d^10^ 6s^2^ 6p^3^), 6 valence electrons for Se (4s^2^ 4p^4^), and 5 for Sb (5s^2^ 5p^3^)] were used, yielding well-converged (to 1 meV/Å) geometries and total energies. A Γ-centered 12 × 12 × 1 k-point mesh was used. The Bi_2_Se_3_ substrate was described using a 6 QL centrosymmetric slab, separated from periodically repeating replicas by thick (> 50 Å) vacuum regions. The experimentally determined atomic positions^59^ and lattice constants (a = 4.143 Å and c = 28.636 Å) were adopted. Sb atoms and the top four atomic layers of the substrate were allowed to relax. β-antimonene/Bi_2_Se_3_ hetero-structures were modelled by placing Sb atoms on both sides of the slab in their most stable geometries^25^. The PBE exchange–correlation functional^60^ was employed with a correction to account for dispersion/vdW forces. The vdW approaches considered include Grimme’s semi-empirical schemes: D2^35^, D3 without damping^61^, and D3-BJ with Becke-Johnson damping^62^; as well as the approach of Tkatchenko-Scheffler (TS)^63^ also with iterative Hirschfeld partitioning (TSH)^64^. Note that for bulk Bi_2_Se_3_, PBE-SOC-D2 yields interlayer distances very close to the experimental one. STM images were simulated via constant local density of states (LDOS) isosurfaces corresponding to the Tersoff-Hamann approximation. STS spectra were obtained by integrating the LDOS inside a 1.5 Å-thick box placed 2.5 Å above the topmost surface atom, and applying a broadening of 0.04 eV.

## Supplementary information

Supplementary information

## Data Availability

The data that support the findings of this study are available from the corresponding authors upon request.
