# Supplementary material for: Topologization of β-antimonene on Bi2Se3 via proximity effects
Source: Sci Rep. 2020 Sep 3;10:14619. doi: 10.1038/s41598-020-71624-4 (PMC7471962; doi:10.1038/s41598-020-71624-4)
Supplement: Supplementary file 1 — Supplementary information [file 41598_2020_71624_MOESM1_ESM.docx]

**Supplementary Information for**

**Topologization of β-antimonene on Bi_2_Se_3_ via proximity effects**

K. Holtgrewe^1^, S. K. Mahatha^2,3🖂^, P. M. Sheverdyaeva^2^, P. Moras^2^, R. Flammini^4^, S. Colonna^4^, F. Ronci^4^, M. Papagno^5^, A. Barla^2^, L. Petaccia^6^, Z. S. Aliev^7^, M. B. Babanly^8^, E. V. Chulkov^9,10, 11,12^, S. Sanna^1^, C. Hogan^4^ and C. Carbone^2^

^1^Institut für Theoretische Physik and Center for Materials Research (LaMa), Justus-Liebig-Universität Gießen, Heinrich-Buff-Ring 16, 35392 Gießen, Germany

^2^Istituto di Struttura della Materia, Consiglio Nazionale delle Ricerche, 34149 Trieste, Italy

^3^Ruprecht Haensel Laboratory, Deutsches Elektronen-Synchrotron DESY, D-22607 Hamburg, Germany

^4^Istituto di Struttura della Materia, Consiglio Nazionale delle Ricerche, Via del Fosso del Cavaliere 100, 00133 Roma, Italy

^5^Dipartimento di Fisica, Università della Calabria, Via P. Bucci, 87036 Arcavacata di Rende (CS), Italy

^6^Elettra Sincrotrone Trieste, Strada Statale 14 km 163.5, 34149 Trieste, Italy

^7^Azerbaijan State Oil and Industry University, AZ1010 Baku, Azerbaijan

^8^Institute Catalysis and Inorganic Chemistry, Azerbaijan National Academy of Science, AZ1143 Baku, Azerbaijan

^9^Centro de Física de Materiales, CFM-MPC, Centro Mixto CSIC-UPV/EHU, Apdo. 1072, 20080 San Sebastián/Donostia, Basque Country, Spain

^10^Donostia International Physics Center (DIPC), P. de Manuel Lardizabal 4, 20018 San Sebastián, Basque Country, Spain

^11^Saint Petersburg State University, 198504 Saint Petersburg, Russia

^12^Institute of Strength Physics and Materials Science, Russian Academy of Sciences, 634021 Tomsk, Russia

^🖂^email: [sanjoymahatha@gmail.com](mailto:sanjoymahatha@gmail.com)

This Supplementary Information contains (1) Band structure of free standing 1BL and 2BL antimonene and parity analysis., (2) Full spin texture of β-antimonene/Bi_2_Se_3_ hetero-structures.

**1. Topological invariants in free-standing β-antimonene**

According to Fu and Kane, the topological *Z_2_* invariant can be computed from the parity of valence bands at the TRIM (time reversal invariant momentum) points in centrosymmetric materials:

${(-1)}^{\nu}= \prod_{i=1}^{TRIM} \delta_{i}$ where $\delta_{i}=\prod_{m=1}^{N} \xi_{2m}^{i}$

Here $\xi$ is the parity eigenvalue and N is the number of occupied bands. Computed parities are reported in Table S1. Since $\upsilon=0$ for both systems, free-standing 1 BL and 2 BL β-antimonene are classed as conventional insulators (CI).

| 1 BL β-antimonene | Parity eigenvalues | Parity product |
| --- | --- | --- |
| $\delta(\Gamma)$ | $+- + + + ; - - -$ | $-$ |
| $3\delta(M)$ | $- + + - - ; + - +$ | $-$ |
| 2 BL β-antimonene | Parity eigenvalues | Parity product |
| $\delta(\Gamma)$ | $+- + - + + + - - - ; + + +$ | $-$ |
| $3\delta(M)$ | $- + - + + - - - + + ; - + +$ | $-$ |

**Table S1. Parity eigenvalues of free-standing β-antimonene.** Semi-colons separate occupied from unoccupied states.

**
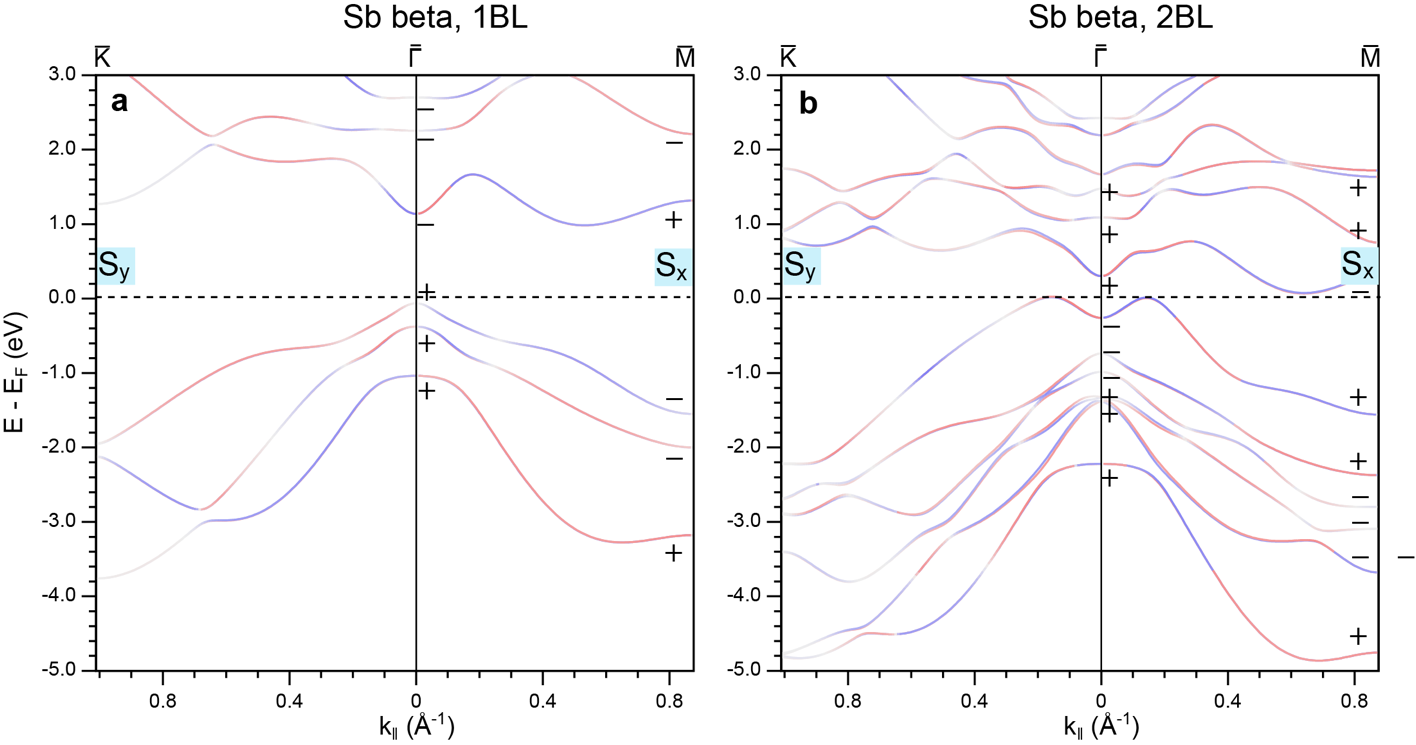
**

**Figure S2. Band structure of free-standing** **a** 1 BL and **b** 2 BL β-antimonene. All bands are doubly degenerate in spin. Spin texture of one band is indicated.

**2. Spin texture of β-antimonene/Bi_2_Se_3_ hetero-structures**

Figures S3 and S4 demonstrate the chiral spin texture for 1 BL and 2 BL β-antimonene on Bi_2_Se_3_.


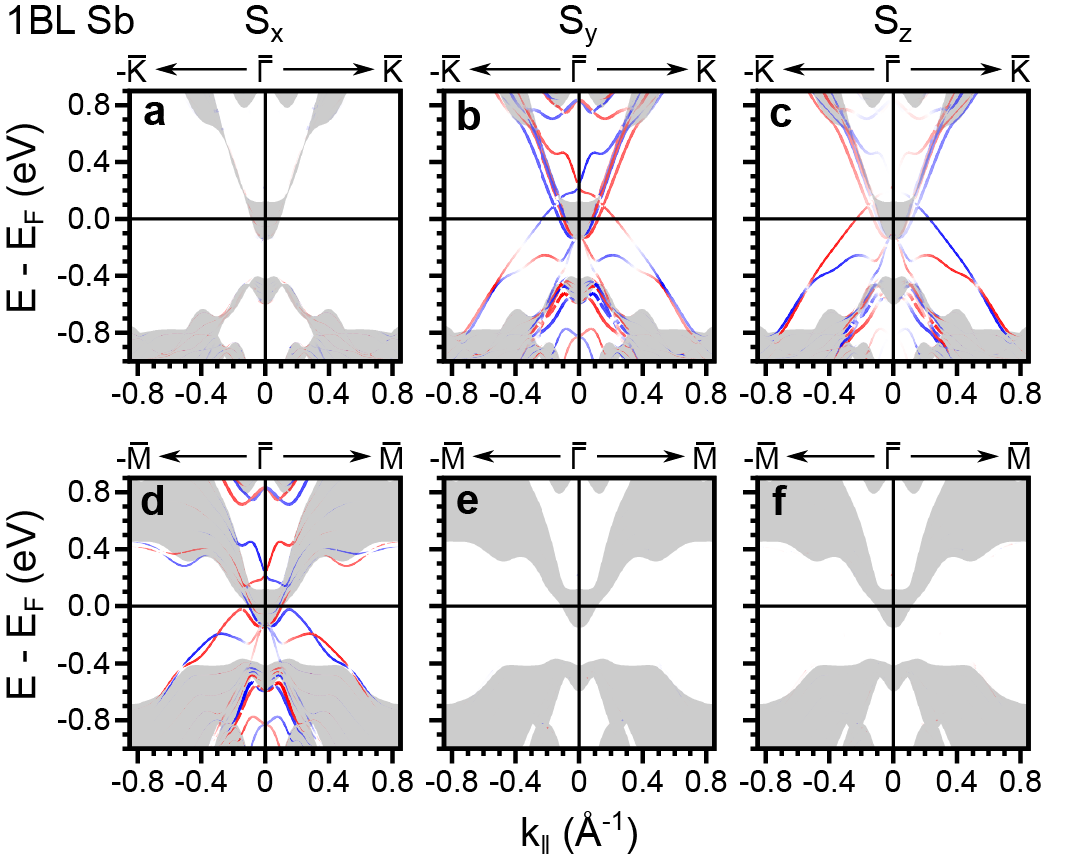


**Figure S3**. **Computed spin textures of 1 BL β-antimonene on Bi_2_Se_3_**. **a,d** S_x_, **b,e** S_y_ and **c,f** S_z_ component along the $\bar{\text{K}}\text{-}\bar{\text{}}\text{-}\bar{\text{K}}$(top row) and $\bar{\text{M}}\text{-}\bar{\text{}}\text{-}\bar{\text{M}}$ (bottom row) directions. Spin up and spin down channels are represented in blue and red, respectively.


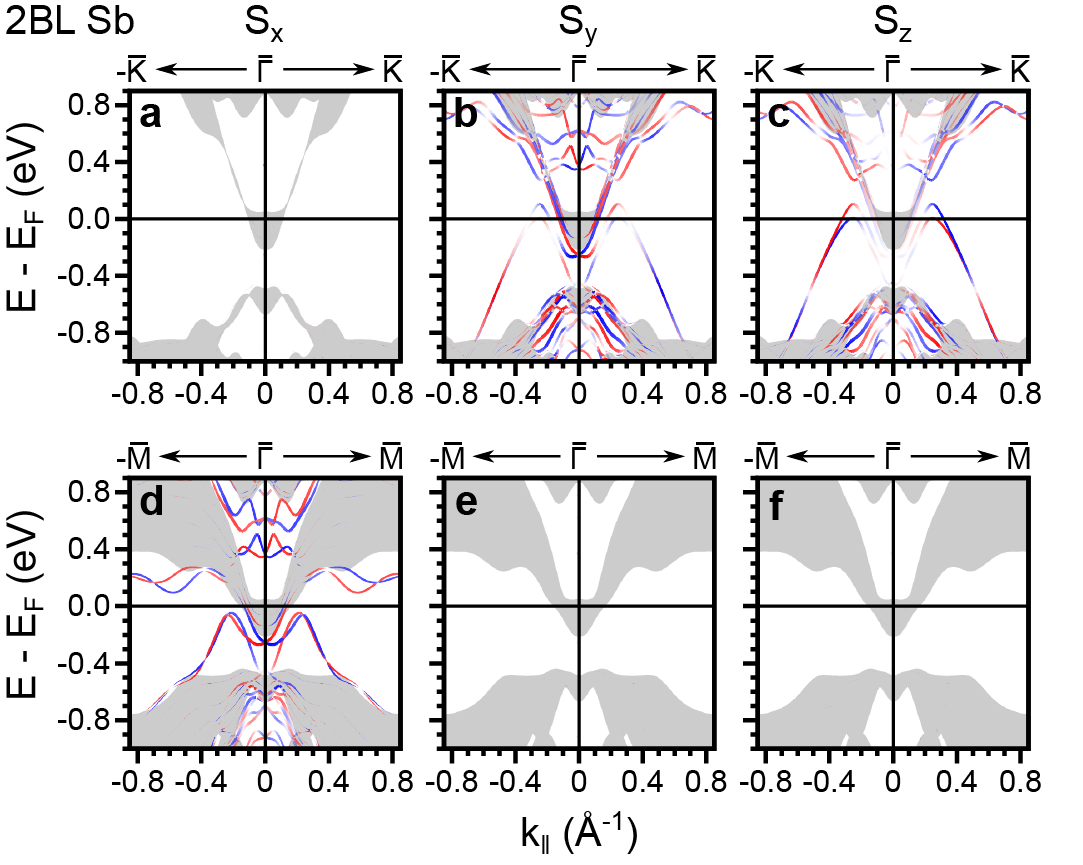


**Figure S4**. **Computed spin textures of 2 BL β-antimonene on Bi_2_Se_3_**. **a,d** S_x_, **b,e** S_y_ and **c,f** S_z_ component along the $\bar{\text{K}}\text{-}\bar{\text{}}\text{-}\bar{\text{K}}$(top row) and $\bar{\text{M}}\text{-}\bar{\text{}}\text{-}\bar{\text{M}}$ (bottom row) directions. Spin up and spin down channels are represented in blue and red, respectively.
